# Perception of optical illusions in ungulates: insights from goats, sheep, guanacos and llamas

**DOI:** 10.1007/s10071-024-01878-2

**Published:** 2024-05-24

**Authors:** Caterina Berardo, Ruben Holland, Alina Schaffer, Alvaro Lopez Caicoya, Katja Liebal, Paola Valsecchi, Federica Amici

**Affiliations:** 1 Department of Chemistry, Life Science and Environmental Sustainability, University of Parma, Parma, Italy; 2 Zoo Leipzig, Leipzig, Germany; 3 Behavioral Ecology Research Group, Institute of Biology, University of Leipzig, Leipzig, Germany; 4 Department of Human Behavior, Ecology and Culture, Max Planck Institute for Evolutionary Anthropology, Leipzig, Germany; 5 Working Group Psychophysiology, Research Institute for Farm Animal Biology (FBN), Dummerstorf, Germany; 6 Department of Clinical Psychology and Psychobiology, Faculty of Psychology, University of Barcelona, Barcelona, Spain; 7 Human Biology and Primate Cognition, Institute of Biology, Faculty of Life Science, Leipzig University, Talstraße 33, 04103 Leipzig, Germany; 8 Department of Comparative Cultural Psychology, Max Planck Institute for Evolutionary Anthropology, Leipzig, Germany

**Keywords:** Müller-Lyer illusion, Delboeuf illusion, Goats, Sheep, Llamas, Guanacos

## Abstract

Optical illusions have long been used in behavioural studies to investigate the perceptual mechanisms underlying vision in animals. So far, three studies have focused on ungulates, providing evidence that they may be susceptible to some optical illusions, in a way similar to humans. Here, we used two food-choice tasks to study susceptibility to the Müller-Lyer and Delboeuf illusions in 17 captive individuals belonging to four ungulate species (*Lama guanicoe, Lama glama, Ovis aries, Capra hircus*). At the group level, there was a significant preference for the longer/larger food over the shorter/smaller one in control trials. Additionally, the whole group significantly preferred the food stick between two inward arrowheads over an identical one between two outward arrowheads in experimental trials of the Müller-Lyer task, and also preferred the food on the smaller circle over an identical one on the larger circle in the experimental trials of the Delboeuf task. Group-level analyses further showed no significant differences across species, although at the individual level we found significant variation in performance. Our findings suggest that, in line with our predictions, ungulates are overall susceptible to the Müller-Lyer and the Delboeuf illusions, and indicate that the perceptual mechanisms underlying size estimation in artiodactyls might be similar to those of other species, including humans.

## Introduction

Susceptibility to optical illusions can result in individuals failing to accurately process visual information and misinterpreting reality in the presence of specific environmental cues (Gregory 1997, 1998). Size illusions, in particular, occur when individuals misperceive the size of an object due to the surrounding background (Shapiro and Todorovic 2017). One of the best-known size illusions is the Müller-Lyer, which occurs when individuals perceive the same line as being longer when in proximity of two arrowheads pointing inward, rather than outward. This illusion is traditionally thought to occur because individuals wrongly extrapolate three-dimensional information from two-dimensional images, perceiving the line between inward-pointing arrowheads as being farther, and thus longer, than the one between outward-pointing arrowheads (Gregory 1963, 1966), although the exact mechanisms explaining this illusion are still discussed (Howe and Purves 2005). Another classical size illusion is the Delboeuf illusion, which occurs when individuals perceive the same circle as being larger if surrounded by a smaller rather than by a larger concentric circle (Coren and Girgus 2022). This illusion is thought to occur because the smaller surrounding circle assimilates the internal circle, making it look larger, whereas the larger surrounding circle contrasts it, making it look smaller than in reality (King 1988).

Several studies have shown that humans are widely susceptible to both the Müller-Lyer and the Delboeuf illusions (Shapiro and Todorovic 2017), although there might be important intra-specific variation linked to cultural, genetic and environmental factors (e.g., exposure to urban environments: (Deręgowski 2017). Since decades, however, researchers also investigate how non-human animals perceive visual illusions (Révész 1924). As the perception of illusions may often occur automatically in humans, and as humans share a similar visual system to other species, it is indeed possible that susceptibility to optical illusions is widespread across animals (Feng et al. 2017). Studying optical illusions in a comparative perspective may be informative for several reasons. Comparing susceptibility to optical illusions across species, for instance, is a non-invasive way to acquire information on the similarity of their visual systems, and to infer whether these systems share a long evolutionary history in common or rather emerged multiple times as the result of convergent evolution (Fujita et al. 2017). If the same susceptibility to a certain illusion is shared by different species, for instance, it is possible to infer that similar neural mechanisms to visually perceive the world were present already in the common ancestor of these species (Feng et al. 2017). In the same line, comparing susceptibility to optical illusions across species is informative to understand the ecological and environmental conditions in which such susceptibility might emerge, and infer the adaptive significance of illusory perceptions (Fujita et al. 2017).

In species other than humans, there is important variation within and across species in susceptibility to optical illusions. Therefore, it is not yet clear to what extent different species share similar perceptual systems, and/or whether such variation rather depends on specific contextual factors (e.g., methodology, demographic characteristics of the study subjects). So far, researchers have used a variety of optical illusions (e.g., Müller-Lyer, Delboeuf, Ebbinghaus, Kanisza, Zöllner) to test different animal taxa, including insects, fish, reptiles and mammals (Mascalzoni and Regolin 2011; Parrish 2021; Qadri and Cook 2015; Santacà et al. 2021; Watanabe 2021). When tested with the Müller-Lyer illusion, most species appear to perceive the same line as being longer when in proximity of two arrowheads pointing inward as compared to outward (Feng et al. 2017), in a way similar to humans, suggesting that susceptibility to size illusions is phylogenetically widespread. However, when exposed to the Delboeuf illusion, only some of the tested species (i.e., *Pan troglodytes:* Parrish and Beran 2014, *Felis catus:* Szenczi et al. 2019, *Pogona vitticeps:* Santacà et al. 2019) appear to perceive the same circle as being larger when surrounded by a smaller than by a larger circle, as humans typically do.

The main aim of this study was to investigate how different ungulate species (i.e., guanacos, *Lama guanicoe*, llamas, *Lama glama*, Skudde sheep, *Ovis aries*, and Damara goats, *Capra hircus*) perceive two optical size illusions: the Müller-Lyer and the Delboeuf illusions. By testing the susceptibility of these yet unstudied ungulate species to two different illusions, we aimed to understand whether susceptibility to size illusions is widespread across ungulates, as their visual systems share a long evolutionary history in common and they might rely on similar neural mechanisms to visually perceive the world. In ungulates, eyes are positioned on the side of the head, providing them with a wide field of view to detect predators (Sugnaseelan et al. 2013), but likely reducing their ability to perceive depth and distance, as the overlap between the visual fields of both eyes is limited (Fowler 2011). While humans have an orbit convergence of 79.3° and a binocular vision field overlap of 140°, ungulates (*Equus caballos, Ovis aries, Bos taurus, Capra hircus*) have an average orbit convergence of 31° ± 6° and an average binocular vision field overlap of 58° ± 5° (Heesy 2004). Although visual acuity may vary across ungulates species (Carroll et al. 2001), vision is considered the dominant sense in ungulates (Fletcher and Lindsay 1968; Lindsay and Fletcher 1968), playing a crucial role in environmental perception (Baldwin 1979, 1981), individual recognition (Davis et al. 1998; Lickliter and Heron 1984; Taylor and Davis 1998) and selection of ecological resources (Arnold 1966; Bazely and Ensor 1989). In ungulates, vision is indeed well-suited to detect movement, identify objects (Caro 1994; Hirata et al. 2019) and distinguish shapes and patterns (Baldwin 1981; Blakeman and Friend 1986; Roitberg and Franz 2004; Schaeffer and Sikes 1971).

In ungulates, to the best of our knowledge, researchers have so far conducted three studies on the perception of optical illusions. First, a bottlenose dolphin (*Tursiops truncatus*), previously trained to select the larger of two circles, later preferred the circle that was surrounded by six smaller rather than larger inducer circles, suggesting susceptibility to the Ebbinghaus illusion (Murayama 2012). Second, horses (*Equus caballus*) that spontaneously preferred a longer over a shorter carrot stick also showed a preference for a carrot stick located between two inward-pointing arrowheads over an identical one located between two outward-pointing arrowheads, suggesting susceptibility to the Müller-Lyer illusion (Cappellato et al. 2020). Third, horses appeared to be susceptible to a Ponzo illusion created by depth cues in photographs (Timney and Keil 1996). Therefore, ungulates appear to be a promising taxon to study optical illusions in a comparative perspective.

Although different ecological characteristics might be linked to the emergence of differences in visual systems (Wasserman et al. 2012), in this study, we hypothesized that all the selected ungulate species would be susceptible to optical illusions, as already shown in dolphins and horses (Cappellato et al. 2020; Murayama 2012), due to a long common evolutionary history of their visual systems. In particular, we predicted that guanacos, llamas, sheep and goats would be susceptible to both the Müller-Lyer (as observed in horses: Cappellato et al. 2020) and the Delboeuf illusions (like bottlenose dolphins, which are susceptible to the similar Ebbinghaus illusion: Murayama 2012). We anticipated that they would prefer the food stick between two inward arrowheads over an identical one between two outward arrowheads in the Müller-Lyer task, and preferring the food surrounded by a smaller circle over an identical one surrounded by a larger circle in the Delboeuf task.

## Methods

*Ethics statement.* The study was carried out in accordance with German national regulations. The experimental procedures were approved by the research coordinator at the Leipzig Zoo, after a risk assessment conducted by the research coordinator, together with the keepers working with the study subjects. The experimental procedures were considered to pose no risk to the animals and to provide them with clear benefits in terms of enrichment. All the animals participated on a completely voluntary basis, and motivation to participate was ensured exclusively by the use of highly preferred food that belonged to their regular diets.

*Study subjects.* We tested 17 subjects belonging to 4 ungulate species (Table 1), including 5 guanacos (*Lama guanicoe*), 3 llamas (*Lama glama*), 5 Skudde sheep (*Ovis aries*) and 4 Damara goats (*Capra hircus*). All subjects were housed with conspecifics at the zoo of Leipzig, in Germany, and were individually recognizable due to differences in their morphological features (e.g., height, size, fur colour). Study subjects included both males and females, and were all adults (i.e., older than one year), except for one sheep younger than one, who was only tested in the first task. The daily diet of all species included hay, which was available ad libitum, and fresh vegetables. None of the study subjects had ever been tested in an optical illusion task before, although all species had occasionally participated in enrichment activities or in other non-invasive experimental tasks (Caicoya et al. 2023; Schaffer et al. 2020, 2021).

**Table 1:** For the Müller-Lyer and Delboeuf tasks, study subjects, species, sex (F for females, M for males), age (in years), performance (i.e. number of correct trials/number of trials) and *p* values of the corresponding binomial test (marked with an asterisk if significant) in the experimental and control conditions

Species	Subject	Sex	Age	Müller-Lyer task		Delboeuf task	
				Experimental: performance, *p*	Control: performance, *p*	Experimental: performance, *p*	Control: performance, *p*
Guanacos	Phibie	F	13	22/24, < 0.001*	37/48, < 0.001*	10/12, 0.039*	18/24, 0.023*
	Lolita	F	5	21/24, < 0.001*	32/48, 0.029*	9/12, 0.146	19/24, 0.007*
	Rike	F	4	21/24, < 0.001*	30/48, 0.111	9/12, 0.146	15/24, 0.308
	Lissitha	F	7	20/24, 0.002*	37/48, < 0.001*	10/12, 0.039*	21/24, < 0.001*
	Maike	F	5	17/24, 0.064	39/48, < 0.001*	9/12, 0.146	19/24, 0.007*
Llamas	Sanchio	M	11	17/24, 0.064	38/48, < 0.001*	6/12, 0.500	20/24, 0.002*
	Krumel	M	7	15/24, 0.308	33/48, 0.0133	9/12, 0.146	19/24, 0.007*
	Flax	M	7	18/24, 0.023*	37/48, < 0.001*	6/12, 0.500	16/24, 0.152
Goats	Bacca	F	6	17/24, 0.064	39/48,0.001*	10/12, 0.039*	16/24, 0.152
	Frangia	F	2	18/24, 0.023*	37/48, < 0.001*	9/12, 0.146	19/24, 0.007*
	Nina	F	1	21/24, < 0.001*	39/48, < 0.001*	9/12, 0.146	20/24, 0.002*
	Zampa	F	1	19/24, 0.007*	33/48, 0.0132	10/12, 0.039*	16/24, 0.152
Sheep	Trilli	F	2	13/24, 0.839	37/48, < 0.001*	9/12, 0.146	19/24, 0.007*
	Bianca	F	4	19/24, 0.007*	39/48, < 0.001*	9/12, 0.146	17/24, 0.064
	Lady	F	3	17/24, 0.064	42/48, < 0.001*	9/12, 0.146	20/24, 0.002*
	Goccia	F	2	20/24, 0.002*	42/48, < 0.001*	9/12, 0.146	20/24, 0.002*
	Fiocco	M	< 1	21/24, < 0.001*	40/48, < 0.001*	–	–

**Fig. 1:**
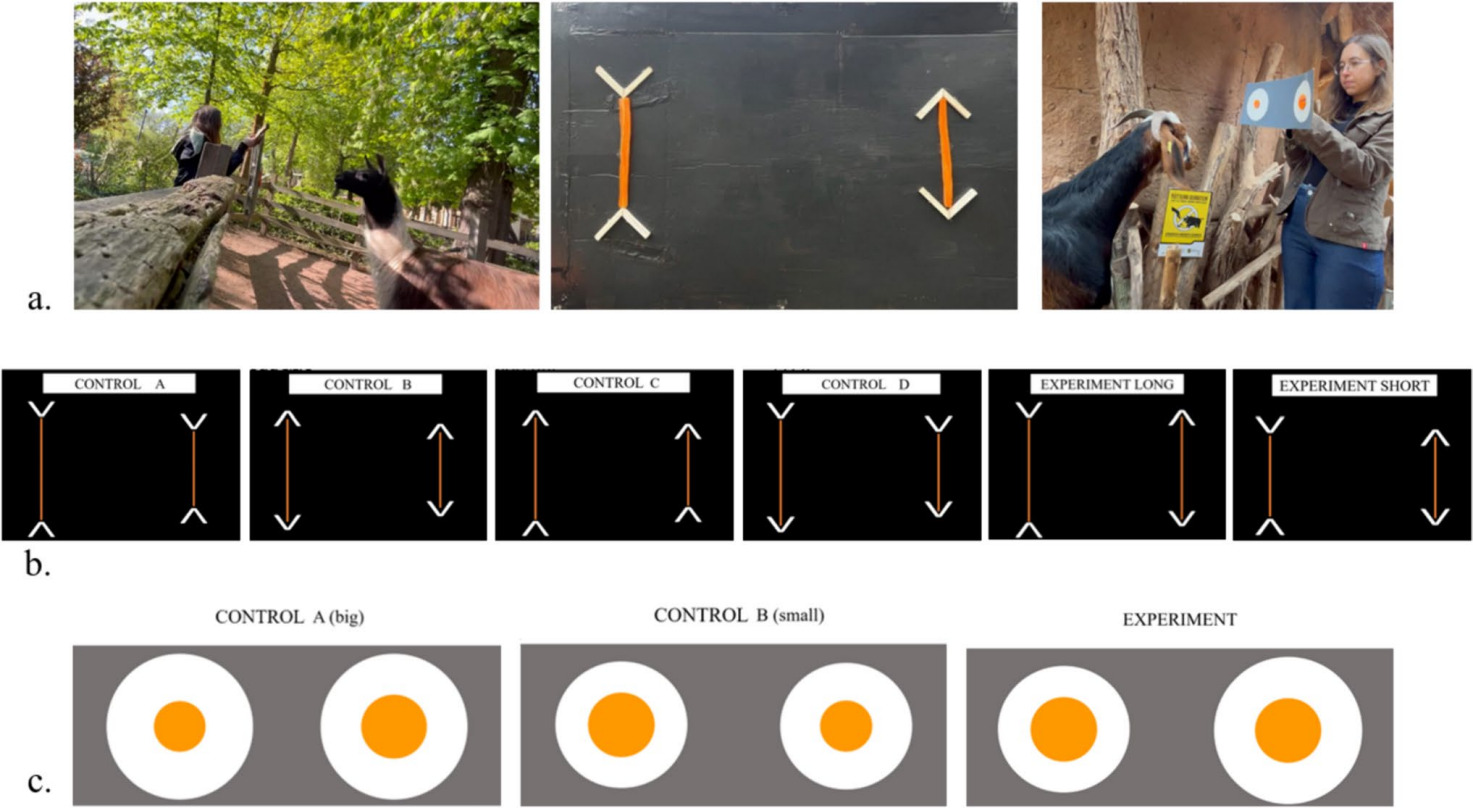
**a** General set-up of the two tasks, including a picture of the board used for the Müller-Lyer task, **b** experimental and control conditions for the Müller-Lyer task and (**c**) for the Delboeuf task

*Materials and procedures*. We administered two tasks: one to test the Müller-Lyer illusion and one to test the Delboeuf illusion. To facilitate comparisons with previous studies, we followed the procedures used in literature with other non-human species (i.e., Müller-Lyer task: Cappellato et al. 2020; Delboeuf task: Parrish and Beran 2014). The tasks were carried out in the outdoor facilities of each species, between 7.30 A.M. and 10.30 A.M., without changing the overall daily routine of the study subjects. The general procedure for both tasks consisted in the experimenter presenting one cardboard to the study subject, either by attaching it to the fence inside the enclosure using a wooden frame (for the guanacos, who were tested in a facility surrounded by 2 m high mesh), or by supporting it with both hands (for the other species, whose facilities were surrounded by a lower fence), so that the cardboard was at the subjects’ eye-level and perpendicular to the ground (Fig. 1a). To avoid separating individuals during the tasks, we waited for one study subject to be alone in proximity of the experimenter and threw a small piece of food at approximately one meter from the cardboard, so that after retrieving the food the subject faced the cardboard frontally. The subject could then approach the cardboard and touch one of the two stimuli attached to the cardboard (see below). As soon as the subject chose one stimulus by touching it with the muzzle or lips, the experimenter allowed the subject to eat the chosen stimulus, while moving the other out of reach. To avoid providing inadvertent cues, the experimenter (i.e., the first author) stood behind the cardboard, between the two stimuli, and always looked straight ahead in front of her. As stimuli, we used familiar food that subjects highly liked and usually received in small quantities. For the Müller-Lyer illusion, we used carrots for all species. For the Delboeuf illusion, which required stimuli with a larger diameter (see below), we used celery for guanacos and lamas (which we painted orange with sweet paprika, to make it visually more salient), and carrots for sheep and goats (which we cut into semicircles that we merged to create round stimuli), as sheep and goats did not like celery. All trials were video-recorded and later coded from the videos.

In the Müller-Lyer task, all the illusions were presented on black cardboards whose measure was adjusted to the size of the study species: 90 × 60 cm for guanacos and llamas, and 60 × 40 cm for sheep and goats. On the right and left halves of the cardboard, we presented two vertical carrot sticks by attaching them by means of toothpicks (Fig. 1b). Since the study species have a blind central area (Sugnaseelan et al. 2013), the distance between the carrot sticks was 60 cm for guanacos and llamas (as for horses: Cappellato et al. 2020), and 40 cm for sheep and goats. Carrot sticks were all equally large and thick (i.e., 1 cm), but their length could vary, being either 20 or 13.5 cm for guanacos and llamas, and 13.5 or 9 cm long for sheep and goats (so that the same length ratio was maintained between longer and shorter carrot sticks for all species). Depending on the condition, we arranged white wooden sticks (7 × 1 × 1 cm for guanacos and llamas, 5 × 1x1 cm for sheep and goats) around the carrot pieces, to form arrowheads on the cardboard (Fig. 1b). All species were tested in six different conditions (Fig. 1b). In the Experimental long condition, we placed two identical longer carrot sticks (i.e., 20 or 13.5 cm, depending on the species) on the cardboard: one with the two arrowheads pointing inward, one outward. The Experimental short condition was identical, except that we used two identical shorter carrot sticks (i.e., 13.5 or 9 cm, depending on the species). The experimental conditions allowed testing whether subjects perceived the Müller-Lyer illusion, perceiving the stick between the two inward arrowheads as being longer than the other stick, and thus preferentially selecting it (Gregory 1997). The four control conditions allowed controlling that subjects reliably selected the longer over the shorter carrot stick when arrowheads were positioned in different ways. In Control condition A, arrowheads pointed inward for both carrots; in Control condition B, they pointed outward; in Control condition C, they pointed upward, and in Control condition D, they pointed downward.

In the Delboeuf task, all the illusions were presented on grey cardboards measuring 45 × 17 cm. On the right and left halves of the cardboard, we painted two white circles that could have a diameter of either 12.5 or 9.5 cm. The distance between the centres of the plates was 30 cm. On the plates, we presented 0.3-cm-thick food circles that could have a diameter of either 4.5 or 3 cm and were attached by means of toothpicks (Fig. 1c). All species were tested in three different conditions (Fig. 1c). In the Experimental condition, we placed two identical larger food items (i.e., 4.5 cm) on a cardboard with a smaller (i.e. 9.5 cm) and a larger (i.e. 12.5 cm) circle. This condition allowed testing whether subjects perceived the Delboeuf illusion, perceiving the food on the smaller circle as being larger than the other one, and thus preferentially selecting it (Coren and Girgus 2022). The two control conditions allowed controlling that subjects reliably distinguished food items of different sizes and consistently selected larger over smaller ones. In Control condition A, we placed a smaller (i.e., 3 cm) and a larger (i.e., 4.5 cm) food item on a cardboard with two identical larger circles (i.e., 12.5 cm); Control condition B was identical, but the smaller and larger food items were placed on a cardboard with two identical smaller circles (i.e., 9.5 cm).

Before being tested, all study subjects went through a habituation phase and a pre-testing phase, to familiarize them with the general procedure and ensure that they spontaneously maximized food intake by selecting the larger of two quantities, respectively. In the habituation phase, subjects were presented with a 6 cm long carrot piece attached to the black cardboard for 6 trials a day, over 2 days, following the general procedure described above. All subjects participated in the habituation and retrieved the food in all the 12 trials. In the pre-testing phase, subjects were simultaneously presented with a longer (i.e., 20 or 13.5 cm, depending on the species) and a shorter carrot (i.e., 13.5 or 9 cm, depending on the species) on a black cardboard without arrowheads, and were tested until reaching criterion (i.e., selecting the longer carrot in at least 10 out of 12 consecutive trials). In both the habituation and the pre-testing phases, we pseudo-randomized and counterbalanced across trials the position of the food. Subjects required on average 16 ± 5 trials to reach criterion (all goats: 12 trials; guanacos: 19 ± 6, range: 12–26 trials; llamas: 21 ± 3, range: 18–25 trials; sheep: 14 ± 3, range: 12–20 trials). After the habituation and pre-testing phases, we administered the Müller-Lyer task (i.e., 12 trials for each of the 6 conditions), presenting up to 6 trials per subject a day (i.e., one for each condition). Then, we administered the Delboeuf task (i.e., 12 trials for each of the 3 conditions), presenting up to 6 trials per subject a day (i.e. two for each condition). In both tasks, we pseudo-randomized and counterbalanced across trials the condition we administered, and the size of the food that was larger or could be perceived as being larger (never presenting it on the same side for more than two trials in a row). As ungulates often show a side bias (Fourie et al. 2021; Leliveld 2019), in case of two consecutive wrong choices on the same side in the control conditions, we administered two additional trials in which only one food item was presented on the cardboard without arrowheads, on the opposite side. As performance in the Experimental conditions could not rely on olfactory cues (as both stimuli had identical size), we included no conditions to test subjects’ use of olfactory cues.

*Data coding and analyses.* For each trial, we coded subject identity, condition, number of trial for each subject (i.e., 1 to 72 for the Müller-Lyer task, 1 to 36 for the Delboeuf task), side chosen (i.e., left or right) and whether the subject chose the side that was longer/larger (in the control conditions) or could appear longer/larger if subjects perceived the illusion (in the experimental conditions). A second observer naïve to the experimental hypothesis re-coded subjects’ choices in 190/1800 trials, from the videos. Inter-observer reliability was excellent (Cohen’s *k*: *k* = 0.97, N = 190, *p* < 0.001).

We ran two generalized linear mixed models (Baayen et al. 2008) in R, using the package glmmTMB (Berry et al. 2017). To this end, we built two datasets, one for each task, entering one line for each subject and trial (N = 1224 for the Müller-Lyer illusion, N = 576 for the Delboeuf illusion). Our binomial response was whether the focal subject chose the side that was/could be perceived as longer (Müller-Lyer task, Model 1) or larger (Delboeuf task, Model 2). In both full models, we entered as test predictors the interaction of condition and species, and the main terms of the interaction. We further included as controls the number of trial and the side chosen, and as random factor the individual identity. These models allowed assessing whether performance varied across conditions, in a different way across species, while controlling for trial number and side chosen. Full models were then compared with likelihood ratio tests to null models that were identical, but did not include test predictors (Dobson and Barnett 2018). In case of a significant difference between the full and the null model, we used the drop1 function to assess which variables were significant. We checked model assumptions, including residual diagnostics and overdispersion, with the “DHARMa” package, and multicollinearity with the “performance” package (maximum variance inflation factors for both models = 1.01), and detected no issues in the models presented.

As our models evidenced no variation in performance across conditions (see Results), we further used Wilcoxon signed-rank tests to assess whether performance in the experimental conditions differed from chance level (0.50). To this end, we built two datasets, one for each task, entering one line for each subject (N = 17 for the Müller-Lyer illusion, N = 16 for the Delboeuf illusion), and specifying the mean proportion of trials in which each subject chose the side that could be perceived as being longer/larger. In this analysis, all species were grouped together, as species had no significant effect in the models (see Results). Finally we ran a binomial test for each individual, to assess whether individual performance in the experimental conditions differed from chance.

## Results

**Fig. 2:**
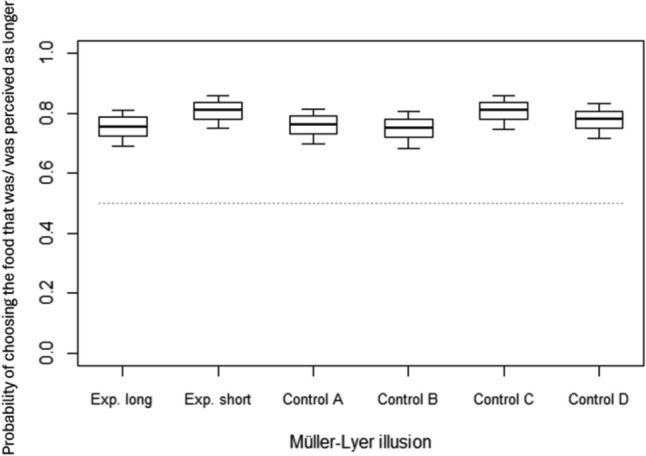
For each condition, mean probability of selecting the food that was longer (in the Control conditions) or was perceived as longer (in the Experimental conditions) in the Müller-Lyer illusion task, across study species. The thick black lines of the box plots represent the mean probabilities for each condition, as estimated by the fitted model (which was like Model 1, but unconditional on all the other factors that were standardized). The ends of the boxes represent the estimated standard errors, and the ends of the whiskers represent the 95% confidence intervals. The grey dotted line represents chance level. Please note that we opted to separately depict all conditions, although there was no significant effect of condition on performance in Model 1

*Müller-Lyer illusion task***.** In the Müller-Lyer task, on average (mean ± SD), subjects chose the carrot that was longer in 75 ± 4% of the control trials (Control condition A: 76 ± 5%; Control condition B: 75 ± 4%; Control condition C: 81 ± 3%; Control condition D: 77 ± 12%), and the carrot that was perceived as longer in 77 ± 7% of the experimental trials (Experimental long condition: 74 ± 6%; Experimental short condition: 80 ± 7%). The full model did not significantly differ from the null model (GLMM, χ^2^ = 27.99, df = 23, *p* = 0.216), suggesting no significant effect of species and condition, neither in interaction nor as main terms, on the probability of choosing the side that was/could be perceived as longer (Table 2). As these results suggest that performance was similar across conditions, and species, we further run a Wilcoxon test at the group level, to assess whether performance in the Experimental and Control conditions differed from chance, regardless of species. Wilcoxon tests showed that, as a group, subjects performed above chance level in the two Experimental conditions (both *p* < 0.001) and in the four Control conditions (all *p* < 0.001), preferring the side that was/was perceived as longer in all conditions (Fig. 2). At the individual level, binomial tests further showed that in the Experimental conditions 11 out of 17 study subjects chose the side that was perceived as longer significantly above chance level, whereas the other 6 subjects preferred the side that was perceived as longer but did not reach significance (Table 1).

**Table 2:** For both models, estimates, standard errors (SE), confidence intervals (CIs), likelihood ratio tests (LRT), degrees of freedom (df), and *p*-values for each test predictor and control (in italics); reference categories are in parentheses

Models, predictors and controls	Estimate	SE	2.5% to 97.5% CI	*df*	*LRT*	*P*
Model 1: Müller-Lyer task						
Intercept	1.38	0.26	0.88 to 1.88	–	–	–
Condition (control B)	− 0.06	0.23	− 0.52 to 0.39	5	4.20	0.521
Condition (control C)	0.28	0.24	− 0.19 to 0.76			
Condition (control D)	0.10	0.24	− 0.36 to 0.56			
Condition (experimental long)	− 0.03	0.23	− 0.49 to 0.43			
Condition (experimental short)	0.29	0.24	− 0.19 to 0.77			
Species (guanacos)	− 0.04	0.19	− 0.41 to 0.34	3	4.61	0.202
Species (lamas)	− 0.22	0.21	− 0.63 to 0.20			
Species (sheep)	0.24	0.20	− 0.14 to 0.63			
Trial number	− 0.05	0.02	− 0.09 to − 0.01	1	6.77	0.009
Choice.side	0.23	0.14	− 0.04 to 0.50	1	2.72	0.099
Model 2: Delboeuf task						
Intercept	0.80	0.31	0.19 to 1.41	–	–	–
Condition (control B)	0.14	0.25	− 0.34 to 0.62	2	1.03	0.596
Condition (experimental)	− 0.10	0.24	− 0.57 to 0.36			
Species (guanacos)	0.14	0.27	− 0.39 to 0.66	3	1.95	0.584
Species (lamas)	− 0.24	0.29	− 0.80 to 0.33			
Species (sheep)	0.10	0.28	− 0.46 to 0.65			
Trial number	0.03	0.03	− 0.02 to 0.09	1	1.42	0.234
Choice.side	0.24	0.20	− 0.15 to 0.64	1	1.48	0.224

**Fig. 3:**
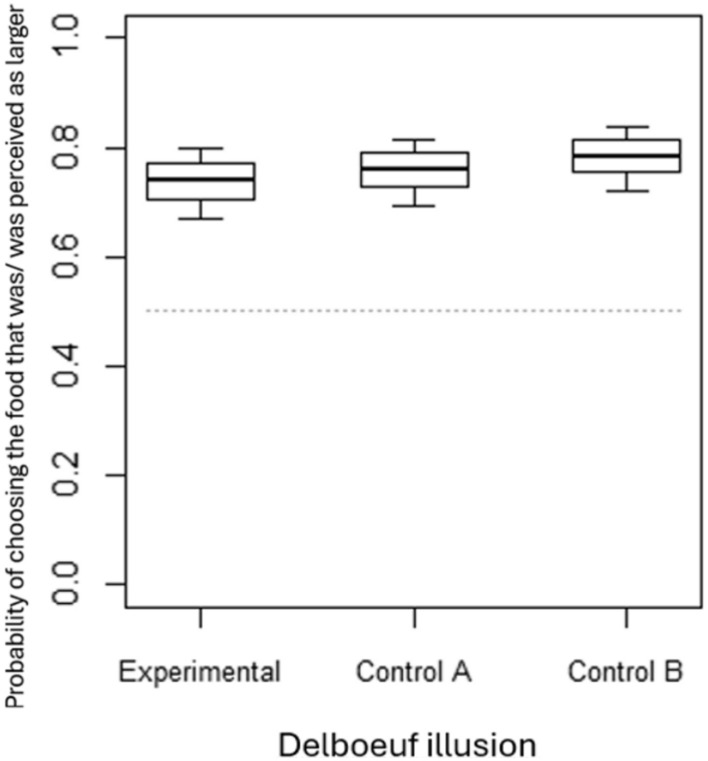
For each condition, mean probability of selecting the food that was larger (in the Control conditions) or was perceived as larger (in the Experimental condition) in the Delboeuf illusion task, across study species. The thick black lines of the box plots represent the mean probabilities for each condition, as estimated by the fitted model (which was like Model 2, but unconditional on all the other factors that were standardized). The ends of the boxes represent the estimated standard errors, and the ends of the whiskers represent the 95% confidence intervals. The grey dotted line represents chance level. Please note that we opted to separately depict all conditions, although there was no significant effect of condition on performance in Model 2

*Delboeuf illusion task*. In the Delboeuf task, on average, subjects chose the food that was larger in 77 ± 3% of the control trials (Control condition A: 75 ± 3%; Control condition B: 78 ± 2%), and the food that was perceived as larger in 73 ± 9% of the experimental trials. The full model did not significantly differ from the null model (GLMM, χ^2^ = 6.60, df = 11, *p* = 0.830), suggesting no significant effect of species and condition on the probability of choosing the side that was/was perceived as larger (Table 2). As above, we thus run a Wilcoxon test at the group level, to assess whether performance in the Experimental and Control conditions differed from chance, regardless of species. Wilcoxon tests showed that, as a group, subjects performed above chance level in the Experimental condition (*p* < 0.001) and in both Control conditions (both *p* < 0.001), preferring the side that was/was perceived as larger in all conditions (Fig. 3). At the individual level, binomial tests further showed that in the Experimental condition 4 out of 16 study subjects chose the side that was perceived as larger significantly above chance level, 10 subjects preferred the side that was perceived as larger but did not reach significance, and 2 subject chose at chance levels (Table 1).

## Discussion

In our study, we tested the susceptibility of subjects belonging to 4 different ungulate species (i.e., guanacos, llamas, sheep and goats) to the Müller-Lyer and Delboeuf illusions. Following the procedures previously used with other species (Cappellato et al. 2020; Parrish and Beran 2014), we found that, at the group level, ungulates perceived both the Müller-Lyer and the Delboeuf illusions, with no significant differences across species.

In the Müller-Lyer task, individuals reliably selected the longer over the shorter stimulus in the control conditions, spontaneously choosing the option that allowed them to maximize food intake. Similarly, at the group level, ungulates reliably selected the food piece between two inward arrowheads over an identical food piece between two outward arrowheads, suggesting that they overall perceived the former as being longer than the latter (Gregory 1997).Therefore, as a group, we found a preference for the food that was or could be perceived as longer above chance level in all conditions, with no variation across species. These results are in line with literature on other mammals, including humans (Shapiro and Todorovic 2017), rhesus macaques (Tudusciuc and Nieder 2010), capuchin monkeys (Suganuma et al. 2007), dolphins (Murayama 2012) and horses (Cappellato et al. 2020;), and on other species of vertebrates (Feng et al. 2017; Pecunioso et al. 2020; Santacà et al. 2021) Fare clic o toccare qui per immettere il testo.. Moreover, these results suggest that susceptibility to this size illusion is widespread across vertebrates, likely because their visual systems share a long evolutionary history in common and may rely on similar neural mechanisms to visually perceive the world. Our results were largely confirmed also at the individual level, with all subjects preferentially selecting the food between two inward arrowheads in the experimental conditions, and 11 out of 17 subjects doing it significantly above chance. On a side note, susceptibility to the Müller-Lyer illusion (but not to the Delboeuf illusion) decreased across trials (Table 2), probably because of a decrease in subjects’ motivation, due to the higher number of trials administered and the gradual emergence of side biases, which is typical of ungulates (Fourie et al. 2021).

In the Delboeuf task, we obtained similar results: individuals reliably selected the larger over the smaller food in the control conditions and, at the group level, ungulates also preferred the food on the smaller circle over an identical one on the larger circle, suggesting that they perceived the former as being larger than the latter. Therefore, as a group, ungulates selected the food that was or could be perceived as larger above chance in all conditions, with no variation across species. These findings suggest that ungulates may be susceptible to the Delboeuf illusion, and are in line with previous findings in humans (see Shapiro and Todorovic 2017) and other species (e.g., chimpanzees: Parrish and Beran 2014, and cats: Szenczi et al. 2019). At the individual level, however, only 4 out of 16 study subjects chose the food on the smaller circle significantly above chance, although no subject preferentially chose the food on the larger circle. As compared to the Müller-Lyer task, the lower number of individuals significantly preferring the food that was perceived as larger may simply depend on the lower power that we had in the second task, where we administered 12 experimental trials per subject, instead of 24. For instance, although Frangia (a goat) chose the side that was perceived as longer/larger in 75% of the experimental trials in both tasks, this resulted in a significant binomial test only in the Müller-Lyer task (Table 1). However, it is also possible that our study subjects were not as susceptible to the Delboeuf illusion as they were to the Müller-Lyer one. In llamas, for instance, two of the three tested individuals chose at chance levels in the Experimental trials, and the third one did not reach significance in his preference for the side that could be perceived as being larger. In the future, larger sample sizes will be needed to understand whether such variation reflects different susceptibility to optical illusions across species or individuals (e.g. due to differences in their perceptual processing strategies: Fuss and Schluessel 2017). At the moment, caution is clearly needed when extrapolating our results, which are unfortunately based on a relatively small sample size.

Our study has several limitations. First, we could only test a limited number of subjects for each species, which might have prevented us from detecting intra-specific variation in how individuals perceive illusions. Although we did not specifically expect any effect of sex on susceptibility to optical illusions, for instance, our study suffered from a strongly biased sex distribution across study species (Table 1). Second, our study only included captive individuals, who may not be good representatives of their wild counterparts. Socio-ecological constraints experienced during ontogeny and extensive exposure to objects and other human artifacts may affect the development of captive individuals (in primates, see Boesch 2007), and perhaps also their susceptibility to optical illusions. In cross-cultural studies on optical illusions, for instance, some researchers have suggested that the socio-ecological challenges experienced might affect human ability to perceive optical illusions (Bremner et al. 2016; Caparos et al. 2012; Segall et al. 1966). Subjects living in a more “carpentered” world (i.e., with frequent right angles and rectangular objects), for instance, may more likely interpret angles in illusions as projections of right angles, and thus more likely perceive some optical illusions (Segall et al. 1966). Our subjects living in zoo enclosures are exposed to a “carpenter world” from their birth and this could have enhanced their susceptibility to visual illusions. Comparisons with wild ungulates will thus be interesting to test the possible effect of environmental conditions on ungulate susceptibility to optical illusions. Similarly, future studies would benefit from the inclusion of a developmental approach, as susceptibility to illusions might change through age also in species other than humans (see e.g., Bánszegi et al. 2021, in cats). Finally, due to time constraints, we also did not include a control condition that is typically used when testing the Delboeuf illusion (Parrish and Beran 2014), in which the smaller food is on the smaller plate and the larger food on the larger plate, which allows excluding the possibility that subjects’ choices depend on the food-to-plate ratio. In future experimental designs, it would be important to include this condition, and also to add further ones to disentangle the relative role played by processes of overestimation (of the food in the smaller circle) and underestimation (of the food in the larger circle) in individuals that perceive the Delboeuf illusion.

In conclusion, our study provides evidence that different ungulate species perceive two classical optical illusions, the Müller-Lyer and Delboeuf illusions, in a way similar to humans and other species, despite important inter-individual variation, especially regarding the Delboeuf illusion. Overall, these results suggest that susceptibility to size illusions is widespread across ungulates, and that the visual systems of our study species might share a long evolutionary history, as the mechanisms evolved for visual perception are similarly deceived by the presence of specific visual cues (e.g., arrowheads, circles; Feng et al. 2017; Fujita et al. 2017). However, only the inclusion of more species will definitely show whether these similarities are the result of convergent evolution (see Fujita et al. 2017, for a discussion). Over the course of millions of years, ungulates have played an essential role in human life, in agricultural settings (Pascual-Rico et al. 2021; Reimoser and Putman 2011), for recreational purposes (Yeates and McGreevy 2019), as companions in equine-assisted therapy (White-Lewis 2020), and as food and economic source (Banda and Tanganyika 2021). Understanding their perceptual systems and skills will hopefully contribute to improve their welfare and management in captive and wild settings (Held et al. 2002), and uphold higher ethical standards when managing these species for human purposes.

## Data Availability

Data are available upon reasonable request to the last author.

## References

[CR1] Arnold GW (1966) The special senses in grazing animals. I. Sight and dietary habits in sheep. Aust J Agric Res 17:521. 10.1071/AR9660521

[CR2] Baayen RH, Davidson DJ, Bates DM (2008) Mixed-effects modeling with crossed random effects for subjects and items. J Mem Lang 59:390–412. 10.1016/j.jml.2007.12.005

[CR3] Baldwin BA (1979) Operant studies on shape discrimination in goats. Physiol Behav 23:455–459. 10.1016/0031-9384(79)90043-X 504436 10.1016/0031-9384(79)90043-x

[CR4] Baldwin BA (1981) Shape discrimination in sheep and calves. Anim Behav 29:830–834. 10.1016/S0003-3472(81)80017-6

[CR5] Banda LJ, Tanganyika J (2021) Livestock provide more than food in smallholder production systems of developing countries. Anim Front 11:7–14. 10.1093/af/vfab001 34026310 10.1093/af/vfab001 PMC8127680

[CR6] Bánszegi O, Szenczi P, Urrutia A, Martínez-Byer S, Hudson R (2021) Visual discrimination of size and perception of the Delboeuf illusion in the domestic cat (*Felis silvestris catus*): a developmental disjunction? J Comp Psychol 135:505–515. 10.1037/com0000288 34435838 10.1037/com0000288

[CR7] Bazely DR, Ensor CV (1989) Discrimination learning in sheep with cues varying in brightness and hue. Appl Anim Behav Sci 23:293–299. 10.1016/0168-1591(89)90098-1

[CR8] Berry RB, Brooks R, Gamaldo C, Harding SM, Lloyd RM, Quan SF, Troester MT, Vaughn BV (2017) AASM scoring manual updates for 2017 (version 2.4). J Clin Sleep Med 13:665–666. 10.5664/jcsm.6576 28416048 10.5664/jcsm.6576 PMC5406946

[CR9] Blakeman NE, Friend TH (1986) Visual discrimination at varying distances in spanish goats. Appl Anim Behav Sci 16:279–283. 10.1016/0168-1591(86)90120-6

[CR10] Boesch C (2007) What makes us human (*Homo sapiens*)? The challenge of cognitive cross-species comparison. J Comp Psychol 121:227–240. 10.1037/0735-7036.121.3.227 17696649 10.1037/0735-7036.121.3.227

[CR11] Bremner AJ, Doherty MJ, Caparos S, de Fockert J, Linnell KJ, Davidoff J (2016) Effects of culture and the urban environment on the development of the Ebbinghaus illusion. Child Dev 87:962–981. 10.1111/cdev.12511 27059268 10.1111/cdev.12511

[CR13] Caicoya AL, Schaffer A, Holland R, von Fersen L, Colell M, Amici F (2023) Innovation across 13 ungulate species: problem solvers are less integrated in the social group and less neophobic. Proc Biol Sci 290:20222384. 10.1098/rspb.2022.2384 37015274 10.1098/rspb.2022.2384 PMC10072937

[CR14] Caparos S, Ahmed L, Bremner AJ, de Fockert JW, Linnell KJ, Davidoff J (2012) Exposure to an urban environment alters the local bias of a remote culture. Cognition 122:80–85. 10.1016/j.cognition.2011.08.013 21962721 10.1016/j.cognition.2011.08.013

[CR15] Cappellato A, MilettoPetrazzini ME, Bisazza A, Dadda M, Agrillo C (2020) Susceptibility to size visual illusions in a non-primate mammal (*Equus caballus*). Animals (basel). 10.3390/ani10091673 32957449 10.3390/ani10091673 PMC7552233

[CR16] Caro TM (1994) Ungulate antipredator behaviour: preliminary and comparative data from African bovids. Behaviour 128:189–228. 10.1163/156853994X00262

[CR17] Carroll J, Murphy CJ, Neitz M, Hoeve JN, Neitz J (2001) Photopigment basis for dichromatic color vision in the horse. J vis 1:80–87. 10.1167/1.2.2 12678603 10.1167/1.2.2

[CR18] Coren S, Girgus J (2022) Seeing is deceiving: the psychology of visual illusions, 1st edn. Routledge, London

[CR20] Davis H, Norris C, Taylor A (1998) Wether ewe know me or not: the discrimination of individual humans by sheep. Behav Processes 43:27–32. 10.1016/S0376-6357(97)00082-X 24897637 10.1016/s0376-6357(97)00082-x

[CR21] Deręgowski JB (2017) Cross-cultural studies of illusions. In: Shapiro AG, Todorovic D (eds) The oxford compendium of visual illusions. Oxford University Press, New York, pp 38–53

[CR22] Dobson A, Barnett AG (2018) An introduction to generalized linear models, 4th edn. Chapman and Hall/CRC

[CR23] Feng LC, Chouinard PA, Howell TJ, Bennett PC (2017) Why do animals differ in their susceptibility to geometrical illusions? Psychon Bull Rev 24:262–276. 10.3758/s13423-016-1133-3 27488557 10.3758/s13423-016-1133-3

[CR24] Fletcher IC, Lindsay DR (1968) Sensory involvement in the mating behaviour of domestic sheep. Anim Behav 16:410–414. 10.1016/0003-3472(68)90032-8 5751507 10.1016/0003-3472(68)90032-8

[CR25] Fourie B, Berezina E, Giljov A, Karenina K (2021) Visual lateralization in artiodactyls: a brief summary of research and new evidence on saiga antelope. Laterality 26:106–129. 10.1080/1357650X.2020.1852245 33593226 10.1080/1357650X.2020.1852245

[CR26] Fowler ME (2011) Medicine and Surgery of Camelids, 3rd edn. Wiley, Somerset

[CR27] Fujita K, Nakamura N, Watanabe S (2017) Visual illusion in a comparative perspective. In: Shapiro AG (ed) The oxford compendium of visual illusions. Oxford University Press, Oxford, pp 54–63

[CR28] Fuss T, Schluessel V (2017) The Ebbinghaus illusion in the gray bamboo shark (*Chiloscyllium griseum*) in comparison to the teleost damselfish (*Chromis chromis*). Zoology (jena) 123:16–29. 10.1016/j.zool.2017.05.006 28712674 10.1016/j.zool.2017.05.006

[CR30] Gregory RL (1963) Distorsion of visual space as inappropriate constance scaling. Nature 199:678–680. 10.1038/199678a0 14074555 10.1038/199678a0

[CR31] Gregory RL (1966) Optical illusions. Nature 209:328. 10.1038/209328a0 5915983 10.1038/209328a0

[CR32] Gregory RL (1997) Knowledge in perception and illusion. Philos Trans R Soc Lond B Biol Sci 352:1121–1127. 10.1098/rstb.1997.0095 9304679 10.1098/rstb.1997.0095 PMC1692018

[CR33] Gregory RL (1998) Eye and brain: the psychology of seeing, 5th edn. Princeton University Press, Princeton

[CR34] Heesy CP (2004) On the relationship between orbit orientation and binocular visual field overlap in mammals. Anat Rec A Discov Mol Cell Evol Biol 281:1104–1110. 10.1002/ar.a.20116 15470671 10.1002/ar.a.20116

[CR35] Held S, Mendl M, Laughlin K, Byrne RW (2002) Cognition studies with pigs: Livestock cognition and its implication for production. J Anim Sci 80:E10–E17. 10.2527/animalsci2002.0021881200800ES10003x

[CR36] Hirata M, Arimoto C, Hattori N, Anzai H (2019) Can cattle visually discriminate between green and dead forages at a short distance while moving in the field? Anim Cogn 22:707–718. 10.1007/s10071-019-01268-z 31127432 10.1007/s10071-019-01268-z

[CR37] Howe CQ, Purves D (2005) The Müller-Lyer illusion explained by the statistics of image-source relationships. Proc Natl Acad Sci U S A 102:1234–1239. 10.1073/pnas.0409314102 15657142 10.1073/pnas.0409314102 PMC544622

[CR39] King DL (1988) Assimilation is due to one perceived whole and contrast is due to two perceived wholes. New Ideas Psychol 6:277–288. 10.1016/0732-118X(88)90039-6

[CR40] Leliveld LMC (2019) From science to practice: a review of laterality research on ungulate livestock. Symmetry 11:1157. 10.3390/sym11091157

[CR41] Lickliter RE, Heron JR (1984) Recognition of mother by newborn goats. Appl Anim Behav Sci 12:187–192. 10.1016/0168-1591(84)90109-6

[CR42] Lindsay DR, Fletcher IC (1968) Sensory involvement in the recognition of lambs by their dams. Anim Behav 16:415–417. 10.1016/0003-3472(68)90033-X 5709770 10.1016/0003-3472(68)90033-x

[CR44] Mascalzoni E, Regolin L (2011) Animal visual perception. Wiley Interdiscip Rev Cogn Sci 2:106–116. 10.1002/wcs.97 26301916 10.1002/wcs.97

[CR45] Murayama T (2012) Relative Size Discrimination and Perception of the Ebbinghaus Illusion in a Bottlenose Dolphin (*Tursiops**truncatus*). Aquat Mamm 38:333–342

[CR47] Parrish AE (2021) Visual illusions: insights from comparative cognition. In: Kuroshima H, Anderson JR (eds) Comparative cognition : commonalities and diversity. Springer, Singapore, pp 15–30

[CR48] Parrish AE, Beran MJ (2014) When less is more: like humans, chimpanzees (*Pan troglodytes*) misperceive food amounts based on plate size. Anim Cogn 17:427–434. 10.1007/s10071-013-0674-3 23949698 10.1007/s10071-013-0674-3 PMC3865074

[CR50] Pascual-Rico R, Morales-Reyes Z, Aguilera-Alcalá N, Olszańska A, Sebastián-González E, Naidoo R, Moleón M, Lozano J, Botella F, von Wehrden H, Martín-López B, Sánchez-Zapata JA (2021) Usually hated, sometimes loved: a review of wild ungulates’ contributions to people. Sci Total Environ 801:149652. 10.1016/j.scitotenv.2021.149652 34438159 10.1016/j.scitotenv.2021.149652

[CR51] Pecunioso A, Santacà M, Petrazzini MEM, Agrillo C (2020) Is the susceptibility to visual illusions related to the relative brain size? Insights from small-brained species. CCBR 15:95–109. 10.3819/CCBR.2020.150003

[CR52] Qadri MAJ, Cook RG (2015) Experimental divergences in the visual cognition of birds and mammals. Comp Cogn Behav Rev 10:73–105. 10.3819/ccbr.2015.100004 26207154 10.3819/ccbr.2015.100004 PMC4507827

[CR53] Reimoser F, Putman R (2011) Impacts of wild ungulates on vegetation: costs and benefits. Ungulate management in Europe, 1st edn. Cambridge University Press, pp 144–191

[CR54] Révész G (1924) Experiments on animal space perception. Brit J Psychol 14:387

[CR55] Roitberg E, Franz H (2004) Oddity learning by African dwarf goats (*Capra hircus*). Anim Cogn 7:61–67. 10.1007/s10071-003-0190-y 13680403 10.1007/s10071-003-0190-y

[CR57] Santacà M, Miletto Petrazzini ME, Agrillo C, Wilkinson A (2019) Can reptiles perceive visual illusions? Delboeuf illusion in red-footed tortoise (*Chelonoidis carbonaria*) and bearded dragon (*Pogona vitticeps*). J Comp Psychol 133:419–427. 10.1037/com0000176 30896231 10.1037/com0000176

[CR59] Santacà M, Agrillo C, MilettoPetrazzini ME (2021) The challenge of illusory perception of animals: the impact of methodological variability in cross-species investigation. Animals. 10.3390/ani11061618 34070792 10.3390/ani11061618 PMC8228898

[CR60] Schaeffer RG, Sikes JD (1971) Discrimination learning in dairy calves. J Dairy Sci 54:893–896. 10.3168/jds.S0022-0302(71)85937-4 5141440 10.3168/jds.S0022-0302(71)85937-4

[CR61] Schaffer A, Caicoya AL, Colell M, Holland R, Ensenyat C, Amici F (2020) Gaze following in ungulates: domesticated and non-domesticated species follow the gaze of both humans and conspecifics in an experimental context. Front Psychol 11:604904. 10.3389/fpsyg.2020.604904 33329278 10.3389/fpsyg.2020.604904 PMC7711155

[CR62] Schaffer A, Caicoya AL, Colell M, Holland R, von Fersen L, Widdig A, Amici F (2021) Neophobia in 10 ungulate species-a comparative approach. Behav Ecol Sociobiol 75:102. 10.1007/s00265-021-03041-0 34177046 10.1007/s00265-021-03041-0 PMC8219784

[CR63] Segall M, Campbell D, Herskovits M (1966) The influence of culture on visual perception. Art Education 23:30. 10.2307/3191488

[CR64] Shapiro AG, Todorovic D (2017) The oxford compendium of visual illusions. Oxford University Press, New York

[CR67] Suganuma E, Pessoa VF, Monge-Fuentes V, Castro BM, Tavares MCH (2007) Perception of the Müller-Lyer illusion in capuchin monkeys (*Cebus apella*). Behav Brain Res 182:67–72. 10.1016/j.bbr.2007.05.014 17586063 10.1016/j.bbr.2007.05.014

[CR68] Sugnaseelan S, Prescott NB, Broom DM, Wathes CM, Phillips CJ (2013) Visual discrimination learning and spatial acuity in sheep. Appl Anim Behav Sci 147:104–111. 10.1016/j.applanim.2013.04.012

[CR69] Szenczi P, Velázquez-López ZI, Urrutia A, Hudson R, Bánszegi O (2019) Perception of the Delboeuf illusion by the adult domestic cat (*Felis silvestris catus*) in comparison with other mammals. J Comp Psychol 133:223–232. 10.1037/com0000152 30394784 10.1037/com0000152

[CR70] Taylor AA, Davis H (1998) Individual humans as discriminative stimuli for cattle (*Bos taurus)*. Appl Anim Behav Sci 58:13–21. 10.1016/S0168-1591(97)00061-0

[CR71] Timney B, Keil K (1996) Horses are sensitive to pictorial depth cues. Perception 25:1121–1128. 10.1068/p251121 8983051 10.1068/p251121

[CR72] Tudusciuc O, Nieder A (2010) Comparison of length judgments and the Müller-Lyer illusion in monkeys and humans. Exp Brain Res 207:221–231. 10.1007/s00221-010-2452-7 20972775 10.1007/s00221-010-2452-7

[CR73] Wasserman EA, Lazareva OF, Shimizu T (2012) How animals see the world: comparative behavior, biology, and evolution of vision. Oxford University Press, New York

[CR74] Watanabe S (2021) Comparative studies on geometric illusions: a review of methods and results. In: Kuroshima H, Anderson JR (eds) Comparative cognition: commonalities and diversity. Springer, Singapore, pp 31–51

[CR75] White-Lewis S (2020) Equine-assisted therapies using horses as healers: a concept analysis. Nurs Open 7:58–67. 10.1002/nop2.377 31871691 10.1002/nop2.377 PMC6917924

[CR76] Yeates J, McGreevy P (2019) Ungulates (Ungulata ). In: Yeates J (ed) Companion animal care and welfare: the UFAW companion animal handbook. Wiley, Hoboken, pp 249–265

